# *cis*-Dichlorido(dimethyl sulfoxide-κ*S*)(*N*,*N*,*N*′,*N*′-tetra­methyl­guanidine-κ*N*′′)platinum(II)

**DOI:** 10.1107/S160053681300130X

**Published:** 2013-01-19

**Authors:** Ivan I. Eliseev, Nadezhda A. Bokach, Matti Haukka, Irina A. Golenya

**Affiliations:** a Department of Chemistry, St Petersburg State University, 198504 Petrodvorets, Russian Federation; b University of Joensuu, Department of Chemistry, PO Box, 111, FI-80101 Joensuu, Finland; c Kiev National Taras Shevchenko University, Department of Chemistry, Volodymyrska Str. 64, 01601 Kiev, Ukraine

## Abstract

In the title compound, *cis*-[PtCl_2_(C_5_H_13_N_3_)(C_2_H_6_OS)], the four-coordinate Pt^II^ atom is bonded to one N atom of the *N*,*N*,*N*′,*N*′-tetra­methyl­guanidine ligand, one dimethyl sulfoxide S atom and two chloride ligands, forming a *cis*-square-planar geometry. The bond lengths and angles of the N—Pt—Cl functionality are typical for imine dichloridoplatinum(II) complexes. The H atom of the imino group is oriented towards the O atom of the sulfoxide group of a neighboring mol­ecule and forms an N—H⋯O hydrogen bond.

## Related literature

For guanidines serving as nucleophiles towards metal-activated nitriles at Pt^II^ and Pt^IV^ atoms, see: Gushchin *et al.* (2007 ▶, 2008 ▶); Tyan *et al.* (2008 ▶). For related structures, see: Bokach *et al.* (2003 ▶); Fairlie *et al.* (1997 ▶); Gonzalez *et al.* (2002 ▶); Makarycheva-Mikhailova *et al.* (2003 ▶). For a description of the Cambridge Structural Database, see: Allen (2002 ▶). For standard bond lengths, see: Allen *et al.* (1987 ▶).
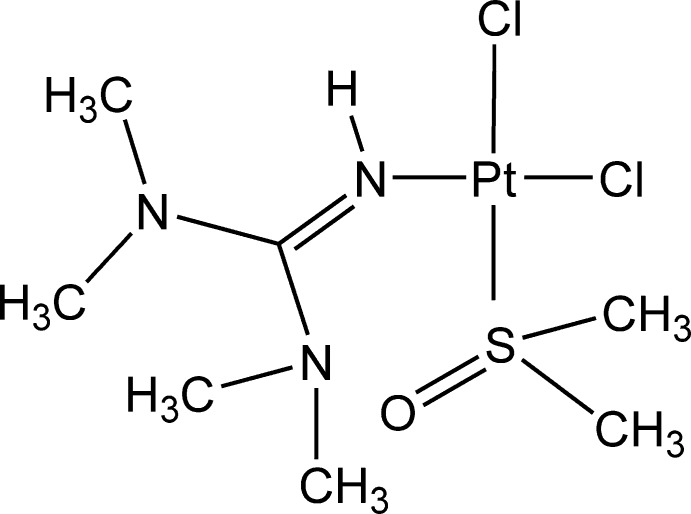


## Experimental

### 

#### Crystal data

[PtCl_2_(C_5_H_13_N_3_)(C_2_H_6_OS)]
*M* *_r_* = 459.30
Monoclinic, 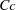

*a* = 10.1577 (5) Å
*b* = 19.1711 (8) Å
*c* = 8.6536 (3) Å
β = 119.304 (2)°
*V* = 1469.51 (11) Å^3^
*Z* = 4
Mo *K*α radiation
μ = 10.04 mm^−1^
*T* = 120 K
0.24 × 0.13 × 0.12 mm

#### Data collection

Nonius KappaCCD diffractometer
Absorption correction: multi-scan (*DENZO*/*SCALEPACK*; Otwinowski & Minor, 1997 ▶) *T*_min_ = 0.151, *T*_max_ = 0.299
12560 measured reflections
3280 independent reflections
3044 reflections with *I* > 2σ(*I*)
*R*_int_ = 0.041

#### Refinement

*R*[*F* ^2^ > 2σ(*F* ^2^)] = 0.023
*wR*(*F* ^2^) = 0.045
*S* = 1.03
3280 reflections
142 parameters
2 restraints
H-atom parameters constrained
Δρ_max_ = 1.49 e Å^−3^
Δρ_min_ = −1.64 e Å^−3^
Absolute structure: Flack (1983 ▶), 1598 Friedel pairs
Flack parameter: 0.008 (6)

### 

Data collection: *COLLECT* (Nonius, 1998 ▶); cell refinement: *DENZO*/*SCALEPACK* (Otwinowski & Minor, 1997 ▶); data reduction: *DENZO*/*SCALEPACK*; program(s) used to solve structure: *SHELXS97* (Sheldrick, 2008 ▶); program(s) used to refine structure: *SHELXL97* (Sheldrick, 2008 ▶); molecular graphics: *DIAMOND* (Brandenburg, 2007 ▶); software used to prepare material for publication: *SHELXL97*.

## Supplementary Material

Crystal structure: contains datablock(s) I, global. DOI: 10.1107/S160053681300130X/hg5281sup1.cif

Structure factors: contains datablock(s) I. DOI: 10.1107/S160053681300130X/hg5281Isup2.hkl

Additional supplementary materials:  crystallographic information; 3D view; checkCIF report

## Figures and Tables

**Table 1:** Hydrogen-bond geometry (Å, °)

*D*—H⋯*A*	*D*—H	H⋯*A*	*D*⋯*A*	*D*—H⋯*A*
N1—H1*N*⋯O1^i^	0.86	2.21	3.021 (5)	159

Symmetry code: (i) 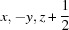.

**Fig. 1.**
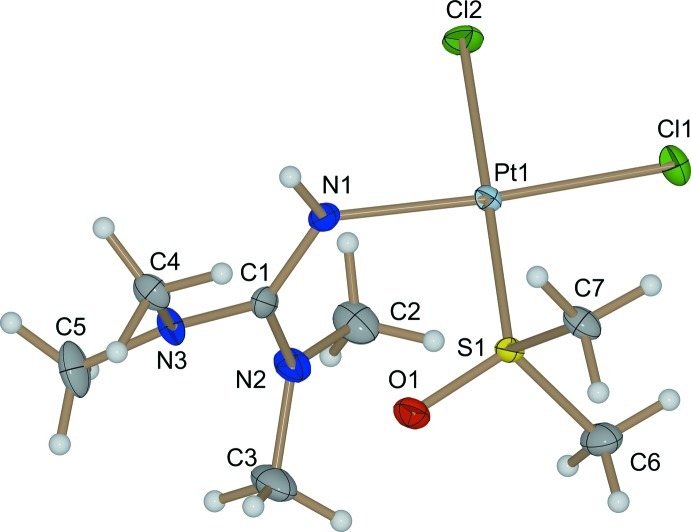
Molecular structure of (I), showing the atom-numbering scheme with displacement ellipsoids drawn at the 50% probability level.

**Fig. 2.**
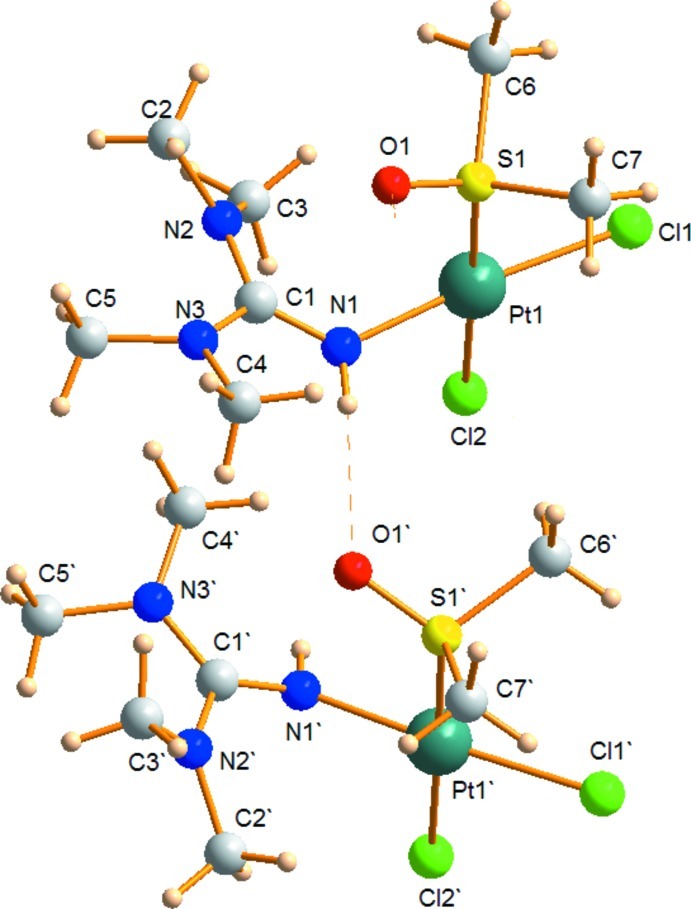
Molecular structure of (I), showing the intermolecular hydrogen bond.

## supplementary crystallographic information

### Comment

As a part of our ongoing investigations on structural features of platinum complexes with guanidines (Gushchin *et al.*, 2007; Gushchin *et al.*, 2008) a new compound, (I), having PtII-bound N,N,N',N'-tetramethylguanidine, has been prepared and herein we report its X-ray crystal and molecular structures.

In the compound, the four-coordinate Pt atom has a distorted cis-square planar geometry where the Pt atom is bonded by one N atom of the N,N,N',N'-tetramethylguanidine ligand and one S atom of the dimethyl sulfoxide and two chlorides in the *cis*-position (Fig. 1 and Table 1). The values of the Pt–Cl bond distances (2.3214 (13) and 2.327 (2) Å) agree well with those of previously characterized platinum(II) chloride compounds (Makarycheva-Mikhailova *et al.*, 2003; Gonzalez *et al.*, 2002). The Pt–N bonds [2.013 (4) Å for Pt–N_imine_] are in accord with those found in *cis-/trans-*[Pt(NH_3_)_2_{NH=C(NH_2_)NMe_2_}] (2.031 (9)/2.020 (3) Å), (Tyan *et al.*, 2008), [Pt{NH=C(NH_2_)NMe_2_}(dien)][SO_3_CF_3_]_2_ (2.018 (7) Å), (Fairlie *et al.*, 1997) and [PtCl_4_{NH=C(NMe_2_)OC(NMe_2_)=NH}] (2.015 (5) Å) (Bokach *et al.*, 2003).

The C=N bond length (C(1)–N(1) 1.316 (6) Å) is equal, within 3σ, to the average C=N double bond distance (1.31 Å) obtained from the Cambridge Crystal Structural Database (Version 5.27; Allen, 2002). The bond lengths C(1)–N(2) and C(1)–N(3) [1.342 (7) and 1.352 (6) Å, respectively] have values closer to a typical C–N single bond [Nsp^2^–Csp^2^ in amides 1.346 (11) Å] (Allen, 1987). In addition, the C=N bond length (1.316 (6) Å) and the C–N bonds lengths (C(1)–N(2) 1.342 (7), C(1)–N(3) 1.352 (6) Å) exhibit values typical, within 3σ, for the (amidine)_2_Pt^II^ complexes, viz. [Pt{NH=C(NH_2_)NMe_2_}(dien)]^2+^, (1.31 (1), 1.394 (8) and 1.33 (1) Å) (Fairlie *et al.*, 1997), and *cis*-/*trans*-[Pt(NH_3_)_2_{NH=C(NH_2_)NMe_2_}] (1.284 (14)/1.288 (5), 1.364 (14)/1.352 (5) and 1.364 (14)/1.341 (5) Å), (Tyan *et al.*, 2008). The H atom of the imino function is oriented towards the O atom of the sulfoxide group of the neighboring molecule forming the intermolecular hydrogen bond (Figure 2, Table 2).

### Experimental

N,N,N',N'-Tetramethylguanidine (13.8 g, 0.12 mmol) was added to K[PtCl_3_(DMSO)] (50.0 mg, 0.12 mmol) in water (1 mL) and the reaction mixture was kept at room temperature for 2 h. The yellow crystalline precipitate were mechanically separated and subjected to the X-ray study. IR (KBr, selected bands, cm–1): 3008 (m, N–H), 1616 (s, C=N), 1134 (s, S=O); 1H NMR (CDCl3, δ, p.p.m.): 4.40 (s, br, 1H, =NH), 3,40 (s, 6H, Me2SO), 3.00 (s, br, 12H, Me2N–); Analyses calculated for C_7_H_19_N_3_Cl_2_OPtS: C 18.31, H 4.17, N 9.15%; found: C 18.05, H 4.15, N 8.59%.

### Refinement

The NH hydrogen atoms was located from the difference Fourier map but constrained to ride on it's parent atom, with U_iso_ = 1.5 U_eq_(parent atom). Other hydrogen atoms were positioned geometrically and were also constrained to ride on their parent atoms, with C—H = 0.98 Å, and U_iso_ = 1.5 U_eq_(parent atom). The highest peak is located 1.49 Å from atom H4B and the deepest hole is located 0.87 Å from atom Pt1.

### Crystal data

**Table:**

[PtCl_2_(C_5_H_13_N_3_)(C_2_H_6_OS)]	*F*(000) = 872
*M**_r_* = 459.30	*D*_x_ = 2.076 Mg m^−^^3^
Monoclinic, *C**c*	Mo *K*α radiation, λ = 0.71073 Å
Hall symbol: C -2yc	Cell parameters from 6239 reflections
*a* = 10.1577 (5) Å	θ = 1.0–27.5°
*b* = 19.1711 (8) Å	µ = 10.04 mm^−^^1^
*c* = 8.6536 (3) Å	*T* = 120 K
β = 119.304 (2)°	Block, pale yellow
*V* = 1469.51 (11) Å^3^	0.24 × 0.13 × 0.12 mm
*Z* = 4	

### Data collection

**Table:**

Nonius KappaCCD diffractometer	3280 independent reflections
Radiation source: fine-focus sealed tube	3044 reflections with *I* > 2σ(*I*)
Horizontally mounted graphite crystal monochromator	*R*_int_ = 0.041
Detector resolution: 9 pixels mm^-1^	θ_max_ = 27.5°, θ_min_ = 3.4°
φ scans and ω scans with κ offset	*h* = −13→13
Absorption correction: multi-scan (*DENZO*/*SCALEPACK*; Otwinowski & Minor, 1997)	*k* = −24→24
*T*_min_ = 0.151, *T*_max_ = 0.299	*l* = −11→11
12560 measured reflections	

### Refinement

**Table:**

Refinement on *F*^2^	Secondary atom site location: difference Fourier map
Least-squares matrix: full	Hydrogen site location: mixed
*R*[*F*^2^ > 2σ(*F*^2^)] = 0.023	H-atom parameters constrained
*wR*(*F*^2^) = 0.045	*w* = 1/[σ^2^(*F*_o_^2^) + (0.*P*)^2^] where *P* = (*F*_o_^2^ + 2*F*_c_^2^)/3
*S* = 1.03	(Δ/σ)_max_ = 0.002
3280 reflections	Δρ_max_ = 1.49 e Å^−^^3^
142 parameters	Δρ_min_ = −1.64 e Å^−^^3^
2 restraints	Absolute structure: Flack (1983), 1598 Friedel pairs
Primary atom site location: structure-invariant direct methods	Flack parameter: 0.008 (6)

### Special details

**Table:**

Geometry. All esds (except the esd in the dihedral angle between two l.s. planes) are estimated using the full covariance matrix. The cell esds are taken into account individually in the estimation of esds in distances, angles and torsion angles; correlations between esds in cell parameters are only used when they are defined by crystal symmetry. An approximate (isotropic) treatment of cell esds is used for estimating esds involving l.s. planes.
Refinement. Refinement of F^2^ against ALL reflections. The weighted R-factor wR and goodness of fit S are based on F^2^, conventional R-factors R are based on F, with F set to zero for negative F^2^. The threshold expression of F^2^ > 2sigma(F^2^) is used only for calculating R-factors(gt) etc. and is not relevant to the choice of reflections for refinement. R-factors based on F^2^ are statistically about twice as large as those based on F, and R- factors based on ALL data will be even larger.

### Fractional atomic coordinates and isotropic or equivalent isotropic displacement parameters (Å^2^)

**Table:**

	*x*	*y*	*z*	*U*_iso_*/*U*_eq_	
Pt1	0.96648 (6)	0.110788 (7)	1.03671 (6)	0.01299 (6)	
Cl1	1.20421 (15)	0.15985 (7)	1.14273 (16)	0.0221 (3)	
Cl2	0.9945 (3)	0.11372 (8)	1.3201 (3)	0.0229 (6)	
S1	0.9352 (2)	0.10943 (7)	0.7678 (3)	0.0133 (5)	
O1	0.7956 (4)	0.07948 (18)	0.6268 (4)	0.0197 (7)	
N1	0.7691 (5)	0.0608 (2)	0.9550 (5)	0.0172 (9)	
H1N	0.7766	0.0172	0.9792	0.026*	
N2	0.5880 (5)	0.1425 (2)	0.7857 (6)	0.0201 (9)	
N3	0.5363 (5)	0.0249 (2)	0.7283 (5)	0.0170 (9)	
C1	0.6341 (6)	0.0760 (3)	0.8237 (6)	0.0162 (10)	
C2	0.6478 (7)	0.1974 (3)	0.9182 (7)	0.0281 (12)	
H2A	0.6875	0.1769	1.0365	0.042*	
H2B	0.5671	0.2305	0.8972	0.042*	
H2C	0.7291	0.2218	0.9105	0.042*	
C3	0.4915 (7)	0.1652 (3)	0.6032 (7)	0.0329 (13)	
H3A	0.4858	0.1281	0.5220	0.049*	
H3B	0.5340	0.2074	0.5807	0.049*	
H3C	0.3901	0.1752	0.5843	0.049*	
C4	0.5856 (6)	−0.0446 (2)	0.7127 (6)	0.0201 (11)	
H4A	0.6924	−0.0432	0.7446	0.030*	
H4B	0.5253	−0.0610	0.5904	0.030*	
H4C	0.5723	−0.0764	0.7926	0.030*	
C5	0.3737 (6)	0.0318 (3)	0.6638 (8)	0.0300 (13)	
H5A	0.3546	0.0749	0.7105	0.045*	
H5B	0.3384	−0.0082	0.7039	0.045*	
H5C	0.3197	0.0334	0.5340	0.045*	
C6	0.9526 (7)	0.1948 (3)	0.7012 (7)	0.0250 (12)	
H6A	0.9526	0.1926	0.5880	0.038*	
H6B	1.0473	0.2157	0.7914	0.038*	
H6C	0.8674	0.2234	0.6872	0.038*	
C7	1.0888 (6)	0.0668 (3)	0.7656 (6)	0.0189 (11)	
H7A	1.0886	0.0174	0.7943	0.028*	
H7B	1.1836	0.0884	0.8537	0.028*	
H7C	1.0791	0.0711	0.6477	0.028*	

### Atomic displacement parameters (Å^2^)

**Table:**

	*U*^11^	*U*^22^	*U*^33^	*U*^12^	*U*^13^	*U*^23^
Pt1	0.01236 (9)	0.01404 (8)	0.01011 (8)	−0.00112 (17)	0.00359 (6)	−0.00035 (14)
Cl1	0.0163 (6)	0.0239 (6)	0.0214 (6)	−0.0052 (5)	0.0056 (5)	−0.0047 (5)
Cl2	0.0297 (13)	0.0281 (12)	0.0108 (10)	−0.0039 (8)	0.0099 (9)	−0.0004 (6)
S1	0.0130 (10)	0.0138 (10)	0.0105 (9)	0.0002 (6)	0.0037 (8)	0.0016 (6)
O1	0.018 (2)	0.0257 (19)	0.0129 (17)	0.0000 (15)	0.0058 (15)	−0.0009 (14)
N1	0.021 (2)	0.016 (2)	0.013 (2)	−0.0045 (17)	0.0072 (18)	0.0003 (16)
N2	0.016 (2)	0.018 (2)	0.022 (2)	0.0048 (18)	0.0057 (19)	0.0029 (18)
N3	0.010 (2)	0.019 (2)	0.020 (2)	−0.0008 (17)	0.0053 (18)	−0.0029 (17)
C1	0.013 (3)	0.022 (3)	0.016 (2)	−0.001 (2)	0.009 (2)	0.002 (2)
C2	0.027 (3)	0.021 (3)	0.030 (3)	0.005 (2)	0.008 (3)	−0.002 (2)
C3	0.026 (3)	0.029 (3)	0.026 (3)	0.002 (3)	−0.001 (3)	0.006 (2)
C4	0.019 (3)	0.019 (3)	0.017 (2)	0.000 (2)	0.006 (2)	−0.006 (2)
C5	0.014 (3)	0.032 (3)	0.044 (4)	−0.003 (2)	0.014 (3)	−0.010 (3)
C6	0.029 (3)	0.021 (3)	0.021 (3)	0.001 (2)	0.009 (2)	0.005 (2)
C7	0.016 (3)	0.019 (3)	0.017 (2)	0.004 (2)	0.004 (2)	−0.002 (2)

### Geometric parameters (Å, º)

**Table:**

Pt1—N1	2.013 (4)	C2—H2C	0.9800
Pt1—S1	2.189 (2)	C3—H3A	0.9800
Pt1—Cl1	2.3214 (13)	C3—H3B	0.9800
Pt1—Cl2	2.327 (2)	C3—H3C	0.9800
S1—O1	1.462 (4)	C4—H4A	0.9800
S1—C7	1.769 (5)	C4—H4B	0.9800
S1—C6	1.773 (5)	C4—H4C	0.9800
N1—C1	1.316 (6)	C5—H5A	0.9800
N1—H1N	0.8556	C5—H5B	0.9800
N2—C1	1.342 (7)	C5—H5C	0.9800
N2—C2	1.452 (7)	C6—H6A	0.9800
N2—C3	1.459 (7)	C6—H6B	0.9800
N3—C1	1.352 (6)	C6—H6C	0.9800
N3—C4	1.451 (6)	C7—H7A	0.9800
N3—C5	1.467 (6)	C7—H7B	0.9800
C2—H2A	0.9800	C7—H7C	0.9800
C2—H2B	0.9800		
			
N1—Pt1—S1	91.13 (12)	N2—C3—H3A	109.5
N1—Pt1—Cl1	175.21 (12)	N2—C3—H3B	109.5
S1—Pt1—Cl1	90.38 (7)	H3A—C3—H3B	109.5
N1—Pt1—Cl2	88.20 (12)	N2—C3—H3C	109.5
S1—Pt1—Cl2	178.66 (11)	H3A—C3—H3C	109.5
Cl1—Pt1—Cl2	90.38 (7)	H3B—C3—H3C	109.5
O1—S1—C7	108.1 (2)	N3—C4—H4A	109.5
O1—S1—C6	107.6 (3)	N3—C4—H4B	109.5
C7—S1—C6	101.2 (3)	H4A—C4—H4B	109.5
O1—S1—Pt1	117.94 (19)	N3—C4—H4C	109.5
C7—S1—Pt1	110.29 (19)	H4A—C4—H4C	109.5
C6—S1—Pt1	110.4 (2)	H4B—C4—H4C	109.5
C1—N1—Pt1	129.5 (3)	N3—C5—H5A	109.5
C1—N1—H1N	111.0	N3—C5—H5B	109.5
Pt1—N1—H1N	115.1	H5A—C5—H5B	109.5
C1—N2—C2	122.2 (4)	N3—C5—H5C	109.5
C1—N2—C3	121.1 (4)	H5A—C5—H5C	109.5
C2—N2—C3	116.1 (4)	H5B—C5—H5C	109.5
C1—N3—C4	122.5 (4)	S1—C6—H6A	109.5
C1—N3—C5	121.4 (4)	S1—C6—H6B	109.5
C4—N3—C5	115.0 (4)	H6A—C6—H6B	109.5
N1—C1—N2	120.9 (5)	S1—C6—H6C	109.5
N1—C1—N3	120.7 (4)	H6A—C6—H6C	109.5
N2—C1—N3	118.4 (4)	H6B—C6—H6C	109.5
N2—C2—H2A	109.5	S1—C7—H7A	109.5
N2—C2—H2B	109.5	S1—C7—H7B	109.5
H2A—C2—H2B	109.5	H7A—C7—H7B	109.5
N2—C2—H2C	109.5	S1—C7—H7C	109.5
H2A—C2—H2C	109.5	H7A—C7—H7C	109.5
H2B—C2—H2C	109.5	H7B—C7—H7C	109.5

### Hydrogen-bond geometry (Å, º)

**Table:**

*D*—H···*A*	*D*—H	H···*A*	*D*···*A*	*D*—H···*A*
N1—H1*N*···O1^i^	0.86	2.21	3.021 (5)	159

Symmetry code: (i) *x*, −*y*, *z*+1/2.

## References

[bb1] Allen, F. H. (2002). *Acta Cryst.* B**58**, 380–388. 10.1107/s0108768102003890 12037359

[bb14] Allen, F. H., Kennard, O., Watson, D. G., Brammer, L., Orpen, A. G. & Taylor, R. (1987). *J. Chem. Soc. Perkin Trans. 2*, pp. S1–19.

[bb2] Bokach, N. A., Pakhomova, T. B., Kukushkin, V. Yu., Haukka, M. & Pombeiro, A. J. L. (2003). *Inorg. Chem.* **42**, 7560–7568. 10.1021/ic034800x 14606852

[bb3] Brandenburg, K. (2007). *DIAMOND* Crystal Impact GbR, Bonn, Germany.

[bb4] Fairlie, D. P., Jackson, W. G., Skelton, B. W., Wen, H., White, A. H., Wickramasinghe, W. A., Woon, T. C. & Taube, H. (1997). *Inorg. Chem.* **36**, 1020–1026. 10.1021/ic961138e 11669664

[bb5] Flack, H. D. (1983). *Acta Cryst.* A**39**, 876–881.

[bb6] Gonzalez, A. M., Cini, R., Intini, F. P., Pacifico, C. & Natile, G. (2002). *Inorg. Chem.* **41**, 470–479. 10.1021/ic010850v 11825073

[bb7] Gushchin, P. V., Bokach, N. A., Luzyanin, K. V., Nazarov, A. A., Haukka, M. & Kukushkin, V. Yu. (2007). *Inorg. Chem.* **46**, 1684–1693. 10.1021/ic061884b 17266300

[bb8] Gushchin, P. V., Tyan, M. R., Bokach, N. A., Revenco, M. D., Haukka, M., Wang, M.-J., Lai, C.-H., Chou, P.-T. & Kukushkin, V. Yu. (2008). *Inorg. Chem.* **47**, 11487–11500. 10.1021/ic702483w 18376821

[bb9] Makarycheva-Mikhailova, A. V., Bokach, N. A., Kukushkin, V. Yu., Kelly, P. F., Gilby, L. M., Kuznetsov, M. L., Holmes, K. E., Haukka, M., Parr, J., Stonehouse, J. M., Elsegood, M. R. J. & Pombeiro, A. J. L. (2003). *Inorg. Chem.* **42**, 301–309. 10.1021/ic025960w 12693210

[bb10] Nonius (1998). *COLLECT* Nonius BV, Delft, The Netherlands.

[bb11] Otwinowski, Z. & Minor, W. (1997). *Methods in Enzymology*, Vol. 276, *Macromolecular Crystallography*, Part A, edited by C. W. Carter Jr & R. M. Sweet, pp. 307–326. New York: Academic Press.

[bb12] Sheldrick, G. M. (2008). *Acta Cryst.* A**64**, 112–122. 10.1107/S0108767307043930 18156677

[bb13] Tyan, M. R., Bokach, N. A., Wang, M.-J., Haukka, M., Kuznetsov, M. L. & Kukushkin, V. Yu. (2008). *Dalton Trans.* pp. 5178–5188. 10.1039/b806862c 18813372

